# Computational Fluid Simulation of Fibrinogen around Dental Implant Surfaces

**DOI:** 10.3390/ijms21020660

**Published:** 2020-01-19

**Authors:** Hiroaki Kitajima, Makoto Hirota, Toshinori Iwai, Kosuke Hamajima, Ryotaro Ozawa, Yuichiro Hayashi, Yasuharu Yajima, Masaki Iida, Toshiyuki Koizumi, Mitomu Kioi, Kenji Mitsudo, Takahiro Ogawa

**Affiliations:** 1 Weintraub Center for Reconstructive Biotechnology, Division of Advanced Prosthodontics, UCLA School of Dentistry, Los Angeles, CA 90095-1668, USA; mhirota@yokohama-cu.ac.jp (M.H.); hamajima.k0329@gmail.com (K.H.); ozrt1021@gmail.com (R.O.); togawa@dentistry.ucla.edu (T.O.); 2 Department of Oral and Maxillofacial Surgery, Graduate School of Medicine, Yokohama City University, 3-9 Fukuura, Kanazawa-ku, Yokohama, Kanagawa 236-0004, Japan; iwai104oams@yahoo.co.jp (T.I.); xyzkaira@yahoo.co.jp (Y.H.); yas.yajima@gmail.com (Y.Y.); masaki.iida0316@gmail.com (M.I.); koizumi-tky@umin.ac.jp (T.K.); kioi@yokohama-cu.ac.jp (M.K.); mitsudo@yokohama-cu.ac.jp (K.M.); 3 Department of Oral and Maxillofacial Surgery/Orthodontics, Yokohama City University Medical Center, 4-57 Urafune, Minami-ku, Yokohama, Kanagawa 232-0024, Japan; 4 Department of Orthodontics, School of Dentistry, Aichi Gakuin University, 2-11 Suemori-dori, Chikusa-ku, Nagoya, Aichi 464-8651, Japan; 5 Division of Prosthodontics and Oral Rehabilitation, Department of Oral Function and Restoration, Graduate School of Dentistry, Kanagawa Dental University, 82 Inaoka-cho, Yokosuka, Kanagawa 238-8580, Japan

**Keywords:** fibrinogen, computational fluid dynamics, dental implant surface, contact angle, hydrophilicity, blood flow, ultraviolet

## Abstract

Ultraviolet treatment of titanium implants makes their surfaces hydrophilic and enhances osseointegration. However, the mechanism is not fully understood. This study hypothesizes that the recruitment of fibrinogen, a critical molecule for blood clot formation and wound healing, is influenced by the degrees of hydrophilicity/hydrophobicity of the implant surfaces. Computational fluid dynamics (CFD) implant models were created for fluid flow simulation. The hydrophilicity level was expressed by the contact angle between the implant surface and blood plasma, ranging from 5° (superhydrophilic), 30° (hydrophilic) to 50° and 70° (hydrophobic), and 100° (hydrorepellent). The mass of fibrinogen flowing into the implant interfacial zone (fibrinogen infiltration) increased in a time dependent manner, with a steeper slope for surfaces with greater hydrophilicity. The mass of blood plasma absorbed into the interfacial zone (blood plasma infiltration) was also promoted by the hydrophilic surfaces but it was rapid and non-time-dependent. There was no linear correlation between the fibrinogen infiltration rate and the blood plasma infiltration rate. These results suggest that hydrophilic implant surfaces promote both fibrinogen and blood plasma infiltration to their interface. However, the infiltration of the two components were not proportional, implying a selectively enhanced recruitment of fibrinogen by hydrophilic implant surfaces.

## 1. Introduction

Biomaterials implanted in the human body will initially interact with blood. Consequently, the exposed biomaterial surface will be covered by host plasma proteins [1]. Protein adsorption determines the capability of material surfaces to attract cells and controls the cascade of events leading to the expression of specific cellular phenotypes necessary for wound healing [2,3]. Fibrinogen and its byproduct, fibrin, play a crucial role in the initial phase of wound healing, particularly during blood clotting, cell recruitment, and angiogenesis.

In titanium implant therapy in the field of dental and orthopedic surgical restoration and reconstruction, bone-to-titanium integration, referred to as osseointegration, is a necessary process for the successful treatment outcome. Osseointegration is the wound healing around titanium, triggered by surface protein adsorption, complement activation, and fabrication of a fibrinogen and fibrin matrix [4]. Several studies have suggested that platelet adhesion and activation are particularly affected by adsorbed fibrinogen via its direct interaction with platelet receptors [5,6,7]. Moreover, fibrinogen is clinically used in fibrin gel complex such as plate-rich fibrin (PRF) and autologous fibrin gel (AFG) to promote bone and soft tissue regeneration around titanium implants [8,9].

The level of hydrophobicity/hydrophilicity influences the bioactivity of the titanium surfaces. As-received titanium surfaces, including the surfaces of titanium-based commercial implant products, are hydrophobic, with their contact angle for water being higher than 70° [10]. Recently, the treatment of titanium with UV light of a particular strength and wavelength, referred to UV photo-functionalization, was discovered as a measure to convert the titanium surfaces to superhydrophilic with a contact angle of 5° or less [11]. The generation of superhydrophilicity is explained by the removal of hydrocarbons that had been unavoidably accumulated on titanium surfaces [12]. The UV treated surfaces show a greater capability of recruiting osteogenic cells and eventually promote osseointegration [13]. However, there is a critical gap in understanding why UV-induced superhydrophilic titanium surfaces have such an excellent cellular affinity.

Due to the significant advancement in computer science, there has been a remarkable progress in numerical simulations. In the field of dentistry, these simulations have been mostly performed for stress analysis using finite element method [14,15,16]. Computational fluid dynamics (CFD) is widely used to simulate fluid flow which cannot be experimentally reproduced. In the fields of neurosurgery and cardiovascular surgery, CFD allows hemodynamic parameters to be assessed non-invasively as an alternative to experiments on living bodies [17,18,19,20,21]. With help from modern mathematical modeling, we believe we are able to simulate the interaction between blood and biomaterial surfaces. There have been no reports on the application of CFD to blood flow analysis around dental implants. This study hypothesizes that the recruitment of fibrinogen to the implant surfaces is influenced by the degrees of hydrophilicity/hydrophobicity of the surface. The objective of this study was to examine the progressive fibrinogen infiltration to the implant surfaces with different degrees of hydrophilicity/hydrophobicity using CFD models. To determine the potential collateral effect by the supposedly enhanced blood plasma flow by hydrophilic implant surfaces, the blood plasma infiltration to the implant interface was also examined. To create a CFD model of titanium implants, an arbitrary dimension of a regular-size dental implant was used.

## 2. Result

### 2.1. Fibrinogen Infiltration to the Interfacial Zone of Implants with Different Contact Angles

**Figure 1:**
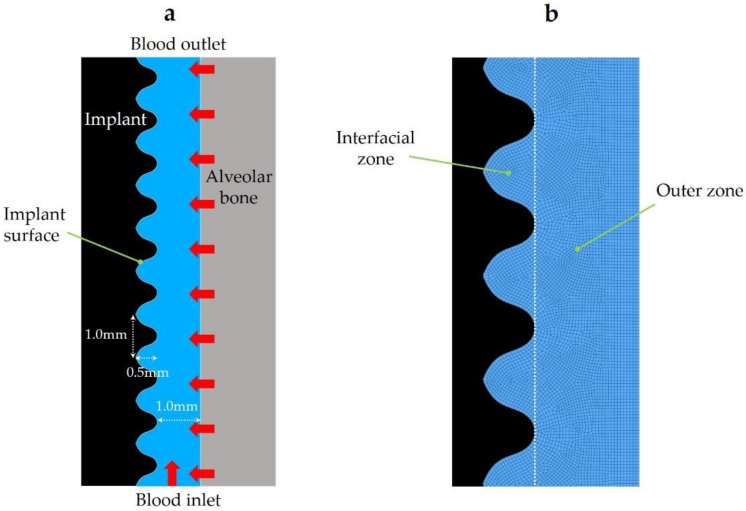
(**a**) Geometrical model and (**b**) computational mesh. The red arrows show the direction of the whole blood flow at the inlets.

We performed the analysis using ANSYS Fluent, a commercially available fluid simulation software package (2019 R1, ANSYS Inc., Canonsburg, PA). We set the contact angle between the implant surface and the blood plasma (CAIS) to a value of 5° (superhydrophilic) or 30° (hydrophilic), 50° or 70° (hydrophobic), and 100° (hydrorepellent), and analyzed the flow of fibrinogen and blood plasma during a period of 3 s after mock placement of an implant in the bone. The analysis time for each implant of different CAIS was approximately 180 min. Movies were made based on the results of the analyses for the implant surfaces with the CAIS of 5° (“The flow of fibrinogen on hydrophilic surface”), 70° (“The flow of fibrinogen on hydrophobic surface”), and 100° (“The flow of fibrinogen on hydrorepellent surface”) using CFD-Post (2019 R1, ANSYS Inc., Canonsburg, PA). These movies can be watched as supplementary materials (see Supplementary Materials). In this study, we divided the geometrical model into two areas, interfacial zone and outer zone, using a vertical line that connects of the peaks of implant threads (Figure 1). In the present study we defined infiltration as the mass of fibrinogen or blood plasma in the interfacial zone for each time step. We focused on the analysis of the interfacial zone, which is thought to be the most important zone for osseointegration. We also used the outer zone as a reference to calculate the fibrinogen infiltration rate and blood plasma infiltration rate. The infiltration rate is defined as the mass of the fibrinogen and blood plasma in the interfacial zone divided by the total amount of fibrinogen and blood plasma present in the fluid zone for each time step, respectively.

**Figure 2:**
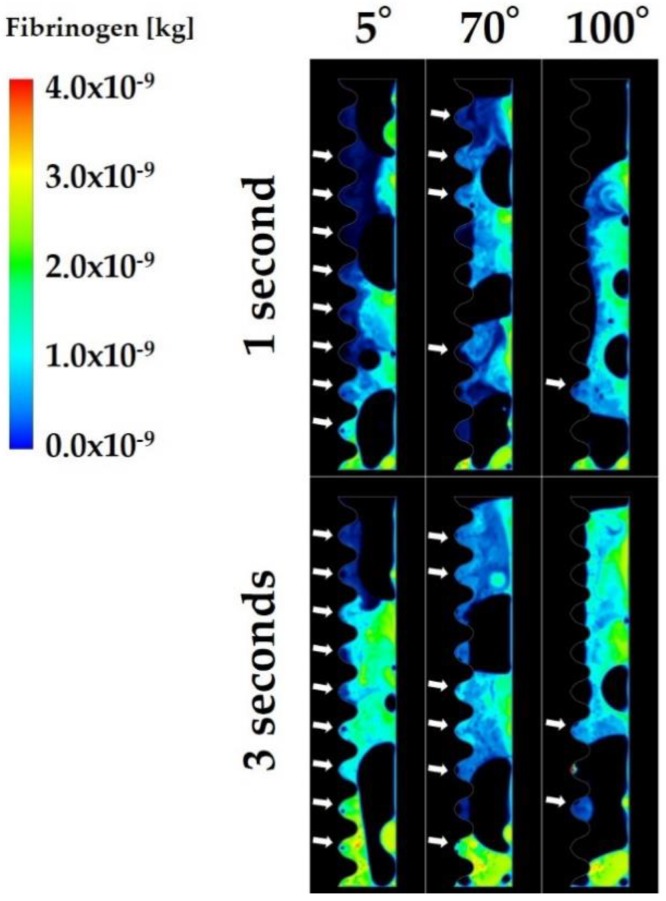
Volume rendering images of the mass of fibrinogen. The white arrows show the threads infiltrated with fibrinogen.

**Figure 3:**
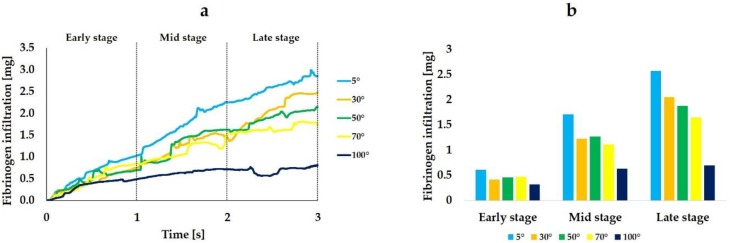
Fibrinogen infiltration (mg) varying with different levels of hydrophilicity or hydrophobicity of the implant surfaces. Line plot graphs (**a**) and integral-based histograms in the early, mid, and late stages (**b**).

Figure 2 shows the volume rendering images indicating the mass of fibrinogen in the fluid zone. The analysis was performed for 3 s, with the first and last seconds defined as early and late stages, respectively. Volume rendering images revealed that more fibrinogen reached the interfacial zone at the implant surface with the CAIS of 5° than at the implant surface with the CAIS of 70° (Figure 2). Most of the implant threads were filled with fibrinogen even after 1 s when the CAIS was 5°. In contrast, the implant threads were largely left blank even after 3 s around the implant of 100° CAIS. Figure 3 is the quantitative presentation of the fibrinogen infiltration. The average of fibrinogen infiltration over time during the 3 s period was 1.6, 1.2, 1.2, 1.1, and 0.6 mg when the CAIS values were 5°, 30°, 50°, 70°, and 100°, respectively. Table 1 shows the time integrals of fibrinogen infiltration and the ratio of the time integral to the value when the CAIS was 5°. Fibrinogen infiltration increased with time from the early, mid, to late stages, regardless of the CAIS (Figure 3a and Table 1).

The more hydrophilic the implant surface was, the higher was the rate of the time-dependent increase. The rate of increase was the highest for the CAIS of 5° and the lowest for 100°. The histogram created for the average fibrinogen infiltration during each of the three stages clearly showed the progressive increase of fibrinogen and its remarkable enhancement when the CAIS was 5° (Figure 3b).

**Figure 4:**
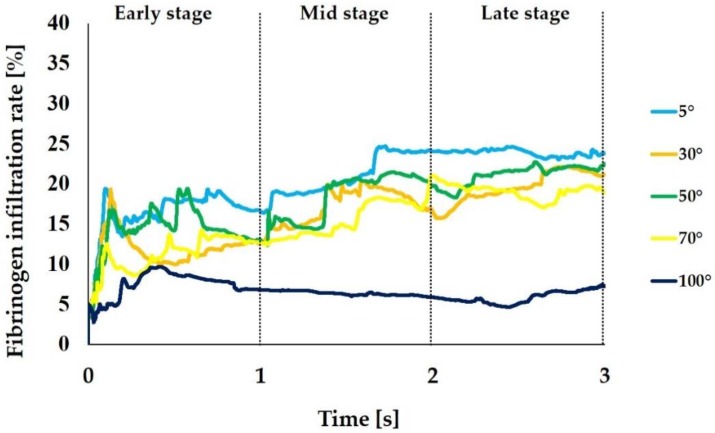
Fibrinogen infiltration rate (%) varying with different levels of hydrophilicity or hydrophobicity of the implant surfaces. The mass of fibrinogen located in the interfacial zone relative to the one in the entire area of the surrounding is expressed in %.

We calculated the infiltration rate for fibrinogen and blood plasma during each time step based on the mass of fibrinogen and blood plasma that were obtained in the analysis. Figure 4 shows the change over time of the fibrinogen infiltration rate. The average infiltration rate from the 3 s analysis was 20.4%, 16.2%, 17.9%, 15.3%, and 6.6% when the CAIS was 5°, 30°, 50°, 70°, and 100°, respectively, thus showing the substantially increased and decreased infiltration rates in the extreme conditions of 5° and 100°, respectively. Particularly, the 5° implant surface showed a substantial increase from the mid-to-late stage, whereas the 100° implant surface, surprisingly showed the progressive decrease with time.

### 2.2. Blood Plasma Infiltration to the Interfacial Zone of Implants with Different Contact Angles

**Figure 5:**
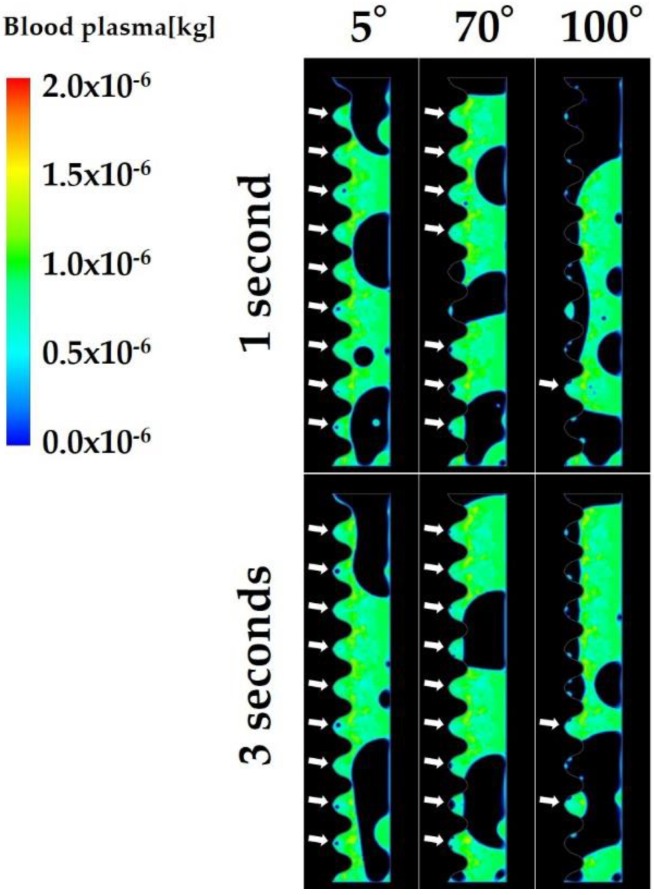
Volume rendering images of the mass of blood plasma. The white arrows show the threads infiltrated with blood plasma.

Figure 5 shows the volume rendering images showing the mass of blood plasma in the fluid zone. Most of the implant threads were filled with blood plasma even after 1 s when the CAIS was 5° and 70°, while most of the implant threads were left blank at 1 s and even after 3 s when the CAIS was 100°.

**Figure 6:**
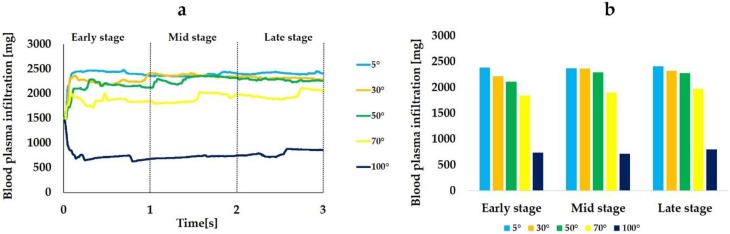
Blood plasma infiltration (mg) varying with different levels of hydrophilicity or hydrophobicity of the implant surfaces. Line plot graphs (**a**) and integral-based histograms in the early, mid, and late stages (**b**).

Figure 6 is a quantitative representation of the blood plasma infiltration. The average of blood plasma infiltration over time during the 3 s period was 2392, 2303, 2229, 1907, and 756 mg when the CAIS values were 5°, 30°, 50°, 70°, and 100°, respectively. Figure 6a shows that the more hydrophilic the surface, the more blood plasma reached to the interfacial zone. However, the time-dependent increase of the infiltration was not observed unlike fibrinogen regardless of the CAIS. The blood plasma infiltration rapidly increases in the beginning of the early stage and reached plateau during the early stage when the CAIS was 5°, 30°, 50°, and 70. While the blood plasma filtration rapidly decreased and remained low even at 3 s when the CAIS was 100° (Figure 6a).

Table 2 shows the time integrals of blood plasma infiltration and the ratio of the time integral to the value when the CAIS was 5° at each time stage. The changes in the blood plasma infiltration among three time stages were smaller than those of fibrinogen (Figure 6b and Table 2).

**Figure 7:**
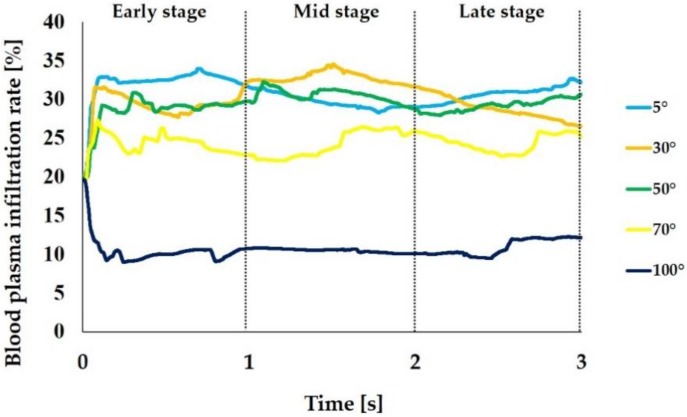
Blood plasma infiltration rate (%) varying with different levels of hydrophilicity or hydrophobicity of the implant surfaces. The mass of blood plasma located in the interfacial zone relative to the one in the entire surrounding area is expressed in %.

Figure 7 shows the blood plasma infiltration rate. The average blood plasma infiltration rate over time during the 3 s was 30.9%, 30.3%, 29.5%, 24.3%, and 10.6% when the CAIS values were 5°, 30°, 50°, 70°, and 100°, respectively. The rate increased in the beginning of the early stage and reached plateau during the early stage when the CAIS was 5°, 30°, 50°, and 70° while it decreased when the CAIS was 100°. Thus, the blood plasma infiltration rate showed the same tendency as the blood plasma infiltration.

### 2.3. Correlation of Fibrinogen Infiltration and Blood Plasma Infiltration Around Implants

**Figure 8:**
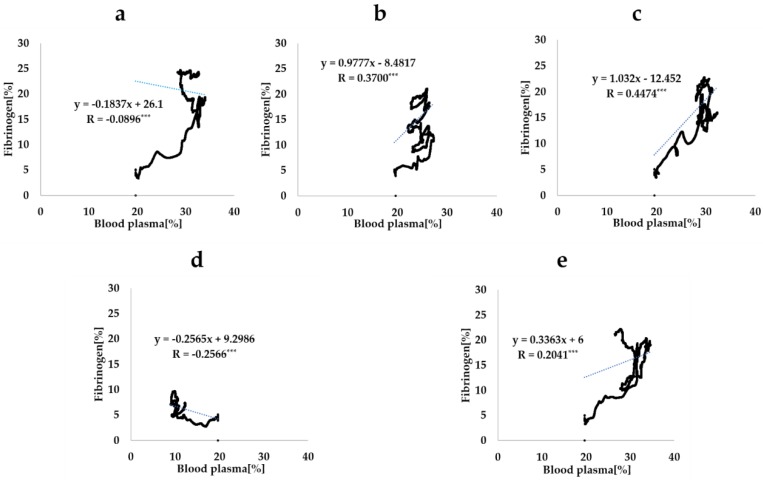
Scatter plots of the fibrinogen infiltration rate and blood plasma infiltration rate. The CAIS values are 0° (**a**), 30° (**b**), 50° (**c**), 70° (**d**), and 100° (**e**). Each dotted line is the regression line calculated from each scatter plot. R indicates the correlation rate (*** *p* < 0.001).

Figure 8 shows the scatter plots of the fibrinogen infiltration rate and blood plasma infiltration rate during the whole duration of the analysis for each CAIS. The correlation coefficients between two rates was −0.0896 (*p* < 0.001), 0.2041 (*p* < 0.001), 0.4474 (*p* < 0.001), 0.3700 (*p* < 0.001), and −0.2566 (*p* < 0.001) when the CAIS values were 5°, 30°, 50°, 70°, and 100°, respectively. There was no linear correlation between the two infiltration rates regardless of the CAIS.

## 3. Discussion

Recent studies found that immediately after processing and regardless of the type of processing, titanium surfaces show a contact angle to water of either 0° or less than 5° [22,23,24,25,26,27]. In the latter case, these surfaces are superhydrophilic. This superhydrophilic nature gradually attenuates, and the surface becomes hydrophobic in 2 weeks as the contact angle changes to over 40°. The contact angle for a 4-week-old acid-etched surface is more than 60° [28]. Aita et al. exposed titanium disks with machined and acid-etched surfaces to UV light, and compared the contact angle to an H_2_O droplet to that of an unexposed disk. The contact angles for the machined and acid-etched surfaces that were not UV-treated were 53.5° and 88.4°, respectively, and those for the UV-treated surfaces were 0° for both cases. The results show that the UV exposure can transform a hydrophobic titanium surface into a superhydrophilic surface [13]. The researchers also showed that the adsorption of albumin and fibronectin, which are proteins in serum, increased significantly in titanium disks treated with UV light compared with untreated counterparts. [29] They hypothesized that UV treatment of dental implants encourages attachment of the osteoblasts through the interaction between the proteins adsorbed on the titanium and the integrins on the cell membranes. However, currently, it is not possible to observe the actual behavior of serum proteins around dental implants in living organisms. Furthermore, since it is difficult to recreate arbitrary contact angles between biomaterial surfaces and the surrounding tissue fluids or H_2_O both in vitro and in vivo, none of the existing reports can verify the impact of hydrophilicity/hydrophobicity of the implant surface on protein adsorption. Therefore, in this study, we used CFD in order to investigate the impact of hydrophilicity/hydrophobicity of the implant surface on the fibrinogen infiltration to the interfacial zone.

The results of the analysis performed in this study showed that as the CAIS decreases, the mass of fibrinogen flowing into the zone nearest to the implant surface (fibrinogen infiltration) increases. The average fibrinogen infiltration during the 3 s period was 1.5 times higher when the CAIS was 5° (hydrophilic) than when it was 70° (hydrophobic). The value was 3.0 times higher when the CAIS was 5° than when it was 100° (hydrorepellent). Furthermore, the change in the time integral of fibrinogen infiltration during each stage plotted in Figure 3 shows that the value increases over time for all values of the CAIS. However, the difference in this value increased with time when the CAIS values were 50°, 70°, and 100° compared to when the CAIS was 5°. When considering the ratio of the mass of fibrinogen to the value when the CAIS was 5° (Table 1), the difference increased over time from 0.17 to 0.27 when the CAIS was 50°, from 0.17 to 0.35 when it was 70°, and from 0.50 to 0.73 when it was 100°. When the CAIS was 30°, the difference in the value compared to the case in which it was 5° changed from 0.33 to 0.19. The value decreased with time. Therefore, as long as the CAIS is under 30°, the adsorbed amount of fibrinogen is expected to increase over time in comparison to when the CAIS is more than 50°.

Similar to the fibrinogen, the mass of blood plasma flowing into the interfacial zone (blood plasma infiltration) increased as the CAIS decreased. The average value during the 3 s period was 1.3 times higher when the CAIS was 5° (hydrophilic) than when it was 70° (hydrophobic). The value was 3.2 times higher when the CAIS was 5° than when it was 100° (hydrorepellent). However, the behavior of the ratio of the blood plasma infiltration to the value when the CAIS was 5° was different from that of fibrinogen. The amplitude of the variation changed from 0.00 to 0.07 when the CAIS was 30°, from 0.03 to 0.11 when it was 50°, from 0.18 to 0.23 when it was 70°, and from 0.67 to 0.70 when it was 100° (Table 2). The change was smaller than the value for fibrinogen (Table 1) for all cases. The change in the time integral of fibrinogen infiltration deviated from the value for blood plasma. These results show that the increase in the fibrinogen infiltration over time was not proportional to the increase in the blood plasma infiltration. Furthermore, the correlation coefficients between the fibrinogen infiltration rate and blood plasma infiltration rate calculated from the plots (Figure 8) show that there was no linear correlation between the two quantities for all time steps during the analysis. Therefore, the results show that the fibrinogen infiltration surrounding the dental implant behaves independently of the blood plasma infiltration. In other words, this result implies that an implant surface with a small CAIS selectively gathers the fibrinogen within the blood plasma towards the interfacial zone.

As shown in Section 4, the flow field was laminar since the Reynolds number was smaller than the boundary value for a turbulent flow (2800). The mass fraction (Yi in Equation (1)) of fibrinogen is governed by advection and diffusion only. Furthermore, the diffusion coefficient of fibrinogen to blood plasma (0.23 × 10^−10^ m^2^/s) was extremely small compared to the time scale (3 s) and spatial scale (1.5 × 10^−3^ m) of the analysis. Therefore, it is clear that the mass fraction is governed more strongly by the advection rather than the diffusion. Accordingly, we surmise that the fibrinogen mass flowing into the interfacial zone increased as the CAIS decreased. This is because a fluid field in which the fibrinogen flowed into the interfacial zone was formed, and the mass was transported due to the advection. UV treatment of titanium dental implants encourages serum protein adsorption due to the change in the electric charge [29]. Researchers have shown that changing a titanium surface from electronegative to electropositive by applying a UV treatment causes adsorption of negatively charged proteins [24,30,31]. Since the effect of electric charge was not considered in this analysis, our study shows that the UV treatment of titanium dental implants encourages protein adsorption. This may be attributed to changes in the fluid field of the blood around the implant that cause protein adsorption as well as the effect of electric charge.

In order to improve our understanding of the phenomenon, it is necessary to perform the analysis for a longer time period, such as several tens of seconds or even minutes. This is because in an actual phenomenon, the whole blood increases its viscosity over time and changes into a blood clot in a few minutes after the placement of implants. However, in that case, it would be necessary to model the changes in viscosity and density of the fluid due to blood coagulation. Furthermore, although we considered only fibrinogen amongst the serum proteins in this study, it is also necessary to analyze the distributions of other major serum proteins, including albumin and fibronectin, which are involved in osseointegration. We anticipate the analysis to be complicated, since the relationships between these proteins such as impact on the diffusion coefficient of each protein, and impact on density and viscosity are unknown.

## 4. Materials and Methods

### 4.1. Geometrical Model

A two-dimensional geometrical model was generated using ANSYS Design Modeler (2019 R1, ANSYS Inc., Canonsburg, PA, USA) to mimic a bone–implant interface (Figure 1). The height and width of the model were 10.0 and 1.5 mm, respectively. The model was comprised of four boundaries and one fluid zone. The blood inlet, alveolar bone, implant surface, and blood outlet were demarcated as the boundaries. Whole blood flowed from the blood inlet and alveolar bone to blood outlet in the analysis. The implant surface had 10 threads. The height and width of each thread were 1.0 and 0.5 mm, respectively.

### 4.2. Mesh Generation

The computational mesh was generated from the geometrical model using ANSYS Meshing (2019 R1, Ansys Inc., Canonsburg, PA, USA; Figure 1). The mesh consisted of 14,610 quadrilateral cells.

### 4.3. Numerical Methods for Blood Flow Simulation

The volumes of fraction (VOF) for blood plasma, red blood cells (RBCs), and the mass fractions for fibrinogen in blood plasma were calculated with the VOF model and species transportation model in ANSYS Fluent (2019 R1, ANSYS Inc., Canonsburg, PA, USA), respectively.

Using the VOF model in ANSYS Fluent, the distribution of the VOF of each fluid in the fluid zone can be analyzed by solving the transport equation of the VOF, the equation for momentum conservation, and the equation for mass conservation. The contact angle between the boundary of the computational mesh and fluids can be defined. In this study, whole blood was assumed to consist of blood plasma and RBCs. Blood plasma and RBCs were set as the primary phase and secondary phase, respectively. They were treated as continuum fluids. We focused on the behavior of fibrinogen and RBCs in the order of millimeters rather than microns. Moreover, the width of the fluid zone (1.5 mm) was significantly larger than the diameter of an RBC, which is approximately 7 μm. The density and viscosity of the RBCs were set to 1125 kg/m^3^ [32] and 0.0050 Pa·S [33], respectively. The density and viscosity of blood plasma are defined in Section 4.4. The continuum surface force model in ANSYS Fluent was used to define the interaction between the blood plasma and RBCs. The value of interfacial tension was set to 0.021 N/m [34].

### 4.4. Numerical Methods for Fibrinogen Flow Simulation

**Figure 9:**
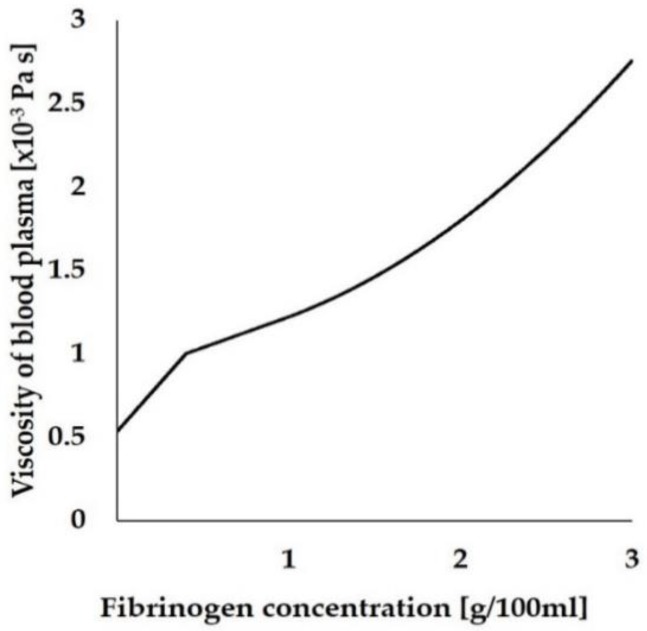
The relationship between viscosity of blood plasma and fibrinogen concentration.

The concentration (mass fraction) of fibrinogen in blood plasma was calculated by the advection-diffusion equation (Equation (1)), as follows:

(1) $$\frac{\partial }{\partial t}\left(\rho_{m}Y_{i}\right)+\nabla \cdot \left(\rho_{m}\vec{v}Y_{i}\right)=-\nabla \cdot \vec{F_{i}},$$

where $Y_{i}$ is the concentration (mass fraction) of the species. The subscript *i* is the species number, and the numbers 0 and 1 indicate fibrinogen and blood plasma, respectively. The mass fraction of the fibrinogen ($Y_{0}$) was calculated by Equation (1). While, the mass fraction of the blood plasma ($Y_{1}$) was calculated as the difference between 1 and $Y_{0}$. As the sum of the $Y_{0}$ and $Y_{1}$ is always 1. $\rho_{m}$ (kg/m^3^) is the density of the mixture of blood plasma and fibrinogen and is described below. $\vec{F_{i}}$ is the diffusion flux of the species. Fick’s law (dilute approximation) was used to express the mass diffusion caused by the mass fraction gradient, and the diffusion flux is expressed using Equation (2) under the law:

(2) $$\vec{F_{i}}=-\rho_{m}D_{i,m}\nabla Y_{i},$$

where $D_{i,m}$ is the diffusion coefficient (m^2^/s) of species *i* in the mixture. Currently, no measurements exist for the diffusion coefficient of fibrinogen to blood plasma. In this study, the diffusion coefficient of fibrinogen to water (0.23 × 10^−10^ m^2^/s [35]) was used as the diffusion coefficient of fibrinogen to blood plasma. The density for whole blood plasma (including fibrinogen, $\rho_{m}$) was defined as a function of $Y_{i}$ according to the volume-weighted mixing law.

(3) $$\rho_{m}=\frac{1}{\sum_{i}\frac{Y_{i}}{\rho_{i}}},$$

where the density of fibrinogen ($\rho_{0}$) and blood plasma (not including fibrinogen, $\rho_{1}$) were set as 1400 [36] and 1025 kg/m^3^ [32], respectively. The viscosity of the whole blood plasma, including fibrinogen ($\mu_{m}$ Pa·S), was defined as a function of fibrinogen concentration, and expressed as Equation (4), which was derived from a previous study [37]. Figure 9 shows the relationship between $\mu_{m}$ and the concentration of fibrinogen.

(4) $$\mu_{m}=\{\begin{array}{c} \left(1.16C+0.53\right)\times 1\times 10^{-3}\;\;\;if\;0\leq C<0.40\\ \left(0.37C+0.85\right)\times 1\times 10^{-3}\;\;\;if\;0.40\leq C<1.00\\ \left(0.19C^{2}+1.03\right)\times 1\times 10^{-3}\;\;\;if\;1.00\leq C \end{array},$$

where C is the fibrinogen concentration (g/100 mL) in blood plasma.

### 4.5. Numerical Conditions

We analyzed the distribution of blood plasma and fibrinogen during the 3-s period following the implantation of the dental implant into the jaw bone using a non-steady analysis. We used a double precision solver and a coupled scheme for the connection between the velocity and pressure. Discretization was performed to a second-order accuracy. The time step size was set to 0.0001 s. Convergence was determined by monitoring the mass (kg) of fibrinogen in the fluid zone. The analysis was determined to have converged when the change in the value for each time step was below 1 × 10^−9^ kg.

The analysis model included two velocity inlets (blood inlet and alveolar bone) and one pressure outlet (blood outlet). A velocity of 0.01 m/s was applied to the velocity inlets. A free stream boundary condition was applied to the outlet. The volume fraction of the RBCs at the velocity inlets was set to 45% (the hematocrit level of a healthy adult), and the volume fraction of the blood plasma was set to 55%. The mass fraction of the fibrinogen at the velocity inlets was set to 0.0029. The value was gained by dividing the reference value of human serum concentration of fibrinogen (300 mg/dL = 3 kg/m^3^) by the density of blood serum (1024 kg/m^3^ [38]). The contact angle between the implant surface and the blood plasma (CAIS) in the VOF model was varied to 5° (Superhydrophilic) or 30° (hydrophilic), 50° or 70° (hydrophobic), and 100° (hydrorepellent). The analysis was performed for each of these conditions. The Reynolds number at the inlet was 6. Since the Reynolds number was smaller than the value at which the flow field transitions to a turbulent flow (namely 2800), the flow field within the fluid zone could be considered to be laminar.

The analysis was performed on a single computer running the Microsoft Windows operating system (Microsoft Windows 10 Professional, Microsoft Corp., Redmond, WA, USA).

## 5. Conclusions

We analyzed the fibrinogen and blood plasma infiltration around a dental implant using CFD. The results show that as the contact angle between the implant surface and the blood plasma decreases, the fibrinogen infiltration increases. Furthermore, the results show that there is no linear correlation between the fibrinogen infiltration rate and blood plasma infiltration rate. This implies that there is a possibility that the hydrophilic implant surface selectively draws fibrinogen from the blood plasma towards the zone nearest to the interface. This study demonstrated the usefulness of CFD for investigating the interaction between blood and dental material surfaces. In order to improve our understanding of this phenomenon, it is necessary to model the blood coagulation and perform the analysis for a longer period of time.

## Figures and Tables

**Table 1:** Time integrals of fibrinogen infiltration and ratio of the time integral to the value when the contact angle between the implant surface and the blood plasma (CAIS) was 5°.

Time Stage	5°	30°	50°	70°	100°
Early stage	0.6 mg (1.00)	0.4 mg (0.67)	0.5 mg (0.83)	0.5 mg (0.83)	0.3 mg (0.50)
Mid stage	1.7 mg (1.00)	1.2 mg (0.71)	1.3 mg (0.76)	1.1 mg (0.65)	0.6 mg (0.35)
Late stage	2.6 mg (1.00)	2.1 mg (0.81)	1.9 mg (0.73)	1.7 mg (0.65)	0.7 mg (0.27)

**Table 2:** Time integrals blood plasma infiltration and ratio of the time integral to the value when the CAIS was 5°.

Time Stage	5°	30°	50°	70°	100°
Early stage	2388 mg (1.00)	2218 mg (0.93)	2114 mg (0.89)	1846 mg (0.77)	742 mg (0.31)
Mid stage	2377 mg (1.00)	2371 mg (1.00)	2295 mg (0.97)	1902 mg (0.80)	721 mg (0.30)
Late stage	2413 mg (1.00)	2322 mg (0.96)	2279 mg (0.94)	1974 mg (0.82)	805 mg (0.33)

## Supplementary Materials

Supplementary materials can be found at https://www.mdpi.com/1422-0067/21/2/660/s1.

## Abbreviations

CAIS	Contact angle between implant surface and blood plasma
CFD	Computational fluid dynamics
